# Large-scale spatial synchrony in red squirrel populations driven by a bottom-up effect

**DOI:** 10.1007/s00442-019-04589-5

**Published:** 2020-01-11

**Authors:** Tytti Turkia, Jussi Jousimo, Juha Tiainen, Pekka Helle, Jukka Rintala, Tatu Hokkanen, Jari Valkama, Vesa Selonen

**Affiliations:** 1 Section of Ecology, Department of Biology, University of Turku, Turku, Finland; 2 Department of Biosciences, Faculty of Biological and Environmental Sciences, University of Helsinki, Helsinki, Finland; 3 Natural Research Institute Finland, Helsinki, Finland; 4 Finnish Museum of Natural History, Helsinki, Finland

**Keywords:** Boreal forest, Population dynamics, *Sciurus vulgaris*, Trophic interactions

## Abstract

Spatial synchrony between populations emerges from endogenous and exogenous processes, such as intra- and interspecific interactions and abiotic factors. Understanding factors contributing to synchronous population dynamics help to better understand what determines abundance of a species. This study focuses on spatial and temporal dynamics in the Eurasian red squirrel (*Sciurus vulgaris*) using snow-track data from Finland from 29 years. We disentangled the effects of bottom-up and top-down forces as well as environmental factors on population dynamics with a spatiotemporally explicit Bayesian hierarchical approach. We found red squirrel abundance to be positively associated with both the abundance of Norway spruce (*Picea abies*) cones and the predators, the pine marten (*Martes martes*) and the northern goshawk (*Accipiter gentilis*), probably due to shared habitat preferences. The results suggest that red squirrel populations are synchronized over remarkably large distances, on a scale of hundreds of kilometres, and that this synchrony is mainly driven by similarly spatially autocorrelated spruce cone crop. Our research demonstrates how a bottom-up effect can drive spatial synchrony in consumer populations on a very large scale of hundreds of kilometres, and also how an explicit spatiotemporal approach can improve model performance for fluctuating populations.

**Electronic supplementary material:**

The online version of this article (10.1007/s00442-019-04589-5) contains supplementary material, which is available to authorized users.

## Introduction

Population dynamics often show positive spatial autocorrelation. That is, changes in abundance in neighbouring populations occur simultaneously and in the same direction, and this synchrony abates with distance (Liebhold et al. 2004). Possible reasons underlying synchrony include dispersal (Ranta et al. 1995; Kendall et al. 2000), regional stochasticity due to environmental factors (Moran effect; Moran 1953; Royama 1992), nomadic predators (Norrdahl and Korpimäki 1996; Korpimäki et al. 2005) and trophic interactions with another species that also shows synchrony (Byholm et al. 2002; Satake et al. 2004; Cattadori et al. 2005). A common cause of synchrony in consumer populations is a spatially autocorrelated food resource. Autocorrelation of the resources can in turn be caused by environmental conditions, which in this context is a form of Moran effect.

Understanding synchronous population dynamics and factors contributing to them can help to better understand core issues in ecology, like what determines abundance and trend of a population. Furthermore, studies on synchronous populations also give valuable information for management of game animals and conservation of threatened species, because synchronous dynamics increase extinction risk of metapopulations (Heino et al. 1997). In these practical implementations, it is essential to be able to predict changes in abundance over time and space. Biotic interactions, such as predation and interspecific competition, affect the distribution of individuals at local scales (Heikkinen et al. 2007) and beyond (Wisz et al. 2013). Thus, a thorough understanding of population dynamics requires that observations from multiple processes are considered simultaneously and the spatiotemporal scale is large enough to allow effects to be seen despite random fluctuations.

Here, we study the range and causes of spatial synchrony between populations of a forest-dwelling boreal mammal, the Eurasian red squirrel (*Sciurus vulgaris*, hereafter red squirrel). The red squirrel is dependent on conifer seeds as the main food resource in boreal forests (Gurnell 1983; Wauters et al. 2008; Selonen et al. 2015). In Fennoscandia, the seeds of the Norway spruce (*Picea abies*, hereafter spruce) are preferred, but seeds of the Scots pine (*Pinus sylvestris*, hereafter pine) are included in the diet as well. Spruce cones mature in autumn and provide a food resource for red squirrels until the next spring, when the seeds fall (Farjon 1990), but the level of the cone crop varies greatly from year to year. When the availability of spruce seeds is low, the red squirrel turns to pine seeds and to the buds of spruce (Rajala and Lampio 1963; Selonen et al. 2016b). Red squirrel numbers increase in spring and summer in response to a good cone crop in preceding autumn and winter (Andrén and Lemnell 1992; Selonen et al. 2015). Studies in southern and central Europe have also found an anticipatory response, whereby red squirrels reproduce more when the coming spruce cone crop in the same autumn will be abundant (Boutin et al. 2006; Wauters et al. 2008). The mast seeding of spruce can be considered as a resource pulse, which can have cascading effects on consumer populations in boreal coniferous forests (Yang et al. 2008).

The most important predators of the red squirrel are the pine marten (*Martes martes*) and birds of prey, especially the northern goshawk (*Accipiter gentilis*, hereafter goshawk; Kenward et al. 1981; Halliwell 1997; Penteriani 1997; Randler 2006). The pine marten is essentially a generalist, but a bulk of the diet consists of rodents, and squirrels comprise a varying proportion of pine marten diet, depending on the relative abundance of red squirrels and the cyclically fluctuating voles (de Marinis and Masseti 1995). Most studies have concluded that the pine marten does not affect the red squirrel on the population level (reviewed in Sheehy and Lawton 2014), but in Scotland, the presence of pine martens did decrease red squirrel density and colonization probability (Halliwell 1997). The goshawk preys on red squirrels regularly (Penteriani 1997), and in some habitats, red squirrels are the most common prey item, especially in winter diet (Kenward et al. 1981; Widén 1987), but red squirrel populations do not seem to be suppressed by the predation (Petty et al. 2003). Dispersal distances of red squirrels in boreal forests are typically 2–4 km (Hämäläinen et al. 2019).

This study aims at assessing spatial and temporal synchrony, temporal trends, and their drivers in red squirrel populations using a long-term snow-track data set that cover most of Finland. We predict that at least close populations are synchronized, and that fluctuations in density follow variations in spruce cone crop. We also account for the density of predators and take into account weather on the day preceding snow-track censuses. Previous studies have suggested that the red squirrel has been declining (Selonen et al. 2010; Turkia et al. 2018a, b), but assessing the population trend of a species with such highly fluctuating population sizes is challenging. Here, we aim to better understand the long-term dynamics by analysing data from 29 years.

## Material and methods

### Red squirrel and pine marten snow-track data

**Fig. 1:**
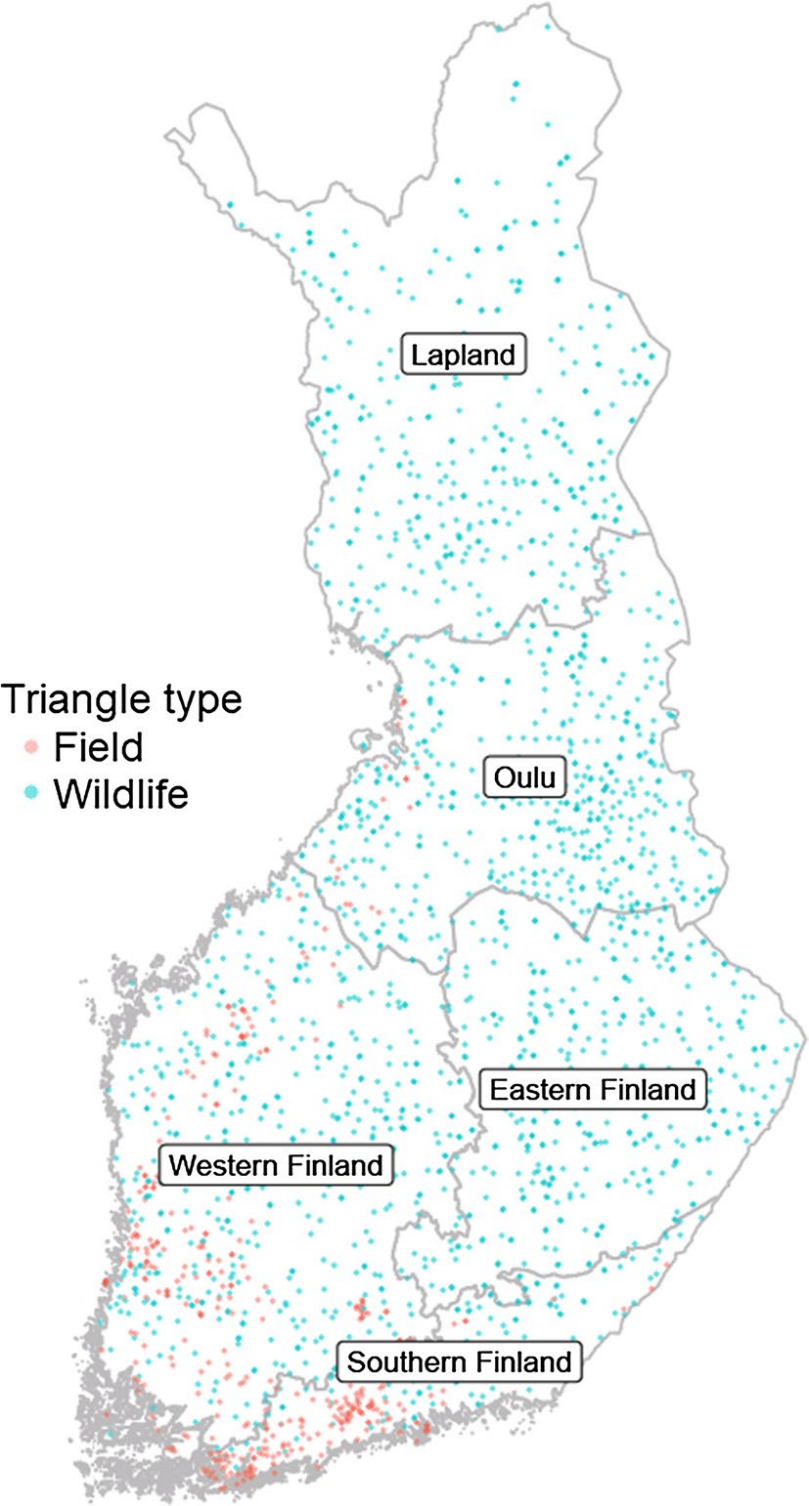
Locations of field triangles (red) and wildlife triangles (blue), and provinces of Finland. Provinces from north to south: Lapland, Oulu, Eastern Finland, Western Finland and Southern Finland

The density of snow tracks was used as a proxy for red squirrel abundance. The number of snow tracks correlates positively with the number of nests and litters of red squirrels (Selonen et al. 2015). Although the red squirrel is an arboreal mammal, they do also commonly move on the ground (even most of their time in late winter) to search for their previously scatter-hoarded food items (Kenward and Tonkin 1986; Wauters et al. 2002) and also to move from tree to tree. Snow-track censuses have been conducted in Finland since 1989 by the former Finnish Game and Fisheries Research Institute (RKTL), which is nowadays part of Natural Resources Institute Finland (Luke). The censuses are based on a network of triangular census lines, known as wildlife triangles and field triangles. Volunteers, usually members of a local hunting club, ski or walk the census line and record all the tracks that cross it and identify the species that made them. The triangles are distributed throughout Finland, excluding the Åland Islands and alpine parts of Lapland (Fig. 1). There are over 1900 wildlife and field triangles together, of which approximately 550 are censused per year. This number has been decreasing during the monitoring scheme, from around 700 per year in the 1990s to around 300 per year in the 2010s. We used data from 1989 to 2017.

Wildlife triangles are 12 km long (4 km per side), and are by definition established in forests. The same triangles are censused every year, regardless of possible management of the forest, thus representing the variety of forest types (Lindén et al. 1996). Field triangles are half the size of wildlife triangles, being 2 km on each side and 6 km in total, and are situated in mosaic-like landscapes, where about half of the census line is in farmland, and the rest of the transect runs through forests, yards and built-up areas (Luke 2018). Field triangles have been established and censused since 1999, and most of them are situated in Western and Southern Finland, where also most of the agriculture is (Fig. 1). We used red squirrel snow-track data from both triangle types. We only used such cases where the census had been made within 1 day after snowfall, or, if the time of the last snowfall was not known, within 1 day and night after last census (collection time = 1 day and night).

The densities of red squirrels and pine martens for each triangle were standardized as the number of snow tracks per triangle and per 1 km, respectively. We also calculated the density of pine marten tracks for the preceding year to test for possible lagged effects between the pine marten and the red squirrel.

### Goshawk data and kernel construction

We modelled goshawk predation pressure at red squirrel census sites, i.e. the triangles, based on goshawk nest locations. We utilized two different goshawk data sets: nest occupancy data and ringing data. The nest occupancy data included locations of nests and their status, occupied or empty. All kinds of signs of goshawk presence (new nest material in old nest site, direct observations of adults, nestlings or eggs in the nest) were considered to indicate occupancy. The ringing data provided locations of nestling ringing, i.e. coordinates of occupied nests. For the most, these data sets included the same information, yet together they held more occupied nests than either data alone, and therefore they were combined. These data were provided by the Finnish ringing centre, which is part of the Finnish Museum of Natural History (LUOMUS). The data are collected by amateur ornithologists who know local forests and their raptor populations well, and therefore we believe that the data represent the true goshawk abundance reasonably well in most parts of the country. However, there is a variation in the search effort over Finland, and also from year to year, because the number of active ornithologists varies. In the used data, there are approximately 2–4 goshawk nests/100 km^2^.

The data on goshawk nest occupancy are collected in summer, whereas snow-track censuses are conducted in late winter, and therefore previous year’s goshawk abundance was used for explaining red squirrel abundance, and lagged effects were calculated from goshawk abundance 2 years before. In other words, the modelled goshawk predation pressure does not describe the situation at the time of the snow-track censuses in January–March. Instead, it reflects the predation pressure during previous summer, when goshawks were at their territory and may have hunted red squirrels that lived, were born within, or tried to disperse into that area. Some of adult goshawks, as well as all juveniles, leave their territory for winter in search of food.

To model the strength of goshawk presence at each wildlife or field triangle, flat-top bivariate Gaussian kernels were calculated around occupied goshawk nests (see Björklund et al. 2016). The value (height) of the kernel at each triangle was used as a proxy for the strength of goshawk predation pressure at that site in the preceding summer. The kernel has values ranging from 0, indicating no goshawks, to 1, indicating strong predation pressure closest to the goshawk nest. The flat-top part of the kernel, 2.5 km, represents this area around the nest where the hawks spend most of the time during the breeding season, affecting their prey animals the most. Beyond this flat-top, the kernel decreases in height towards 0 following the bivariate Gaussian distribution with standard deviation of 4. Because the real impact of goshawks on their prey at different distances from the nests is not known, the flat-top and SD values were chosen from all combinations of SDs 1, 2, 3 and 4 and flat-top values of 500–2500 m on 500 m interval. These combinations were compared using kernel values calculated with each as an explanatory variable for red squirrel snow-track density and by comparing AICs of these models (Björklund et al. 2016). The kernel resulting in lowest model AIC was used. Other models were separated from the best one (i.e. that with 2.5 km flat-top and SD 4) by more than 2 AIC units. The kernel was cut off at a distance of 10 km from the nest. The practical interpretation is that the effect of goshawks on red squirrels within greater radii was deemed negligible. The effect of goshawks was assumed to be highest if the hawks had nested in the summer preceding snow-track census. Also, occupied nests up to 15 years apart from snow-track census were considered to cause a slight predation pressure, because goshawk territories are known to remain occupied for even decades, although goshawks may not reproduce every year (J. Valkama unpubl.). The rate of how the predatory effect of goshawks on red squirrels declines in time since known nest occupancy is not known. We used a 10% decline for every year between nest occupancy and red squirrel snow-track census.

### Spruce cone data

Spruce flowers in spring and the resulting cones mature in autumn and hold their seeds over winter, dropping them in late winter or spring (Farjon 1990). The cone crop in previous years was used as an explanatory variable for red squirrel data, because cone data are collected in autumn (year *y*), whereas the snow-track censuses are made in mid-winter (year *y* + 1). Data on spruce cone numbers per tree in research forest patches were provided by the former Forestry Research Institute Finland (Metla), which has since become part of the Natural Resources Institute Finland (Luke). There are 92 research forests distributed throughout Finland, and both pistillate flowers and cones are counted yearly. Missing values in the data from southern Finland (south of KKJ 7080000 or 63° 50.65′ lat) were replaced with zeros in cone crop failure years (1990, 1994, 1997, 1999, 2001, 2005, 2009, and 2013), because missing values in those years practically imply that the census was not made due to absence of cones (T. Hokkanen unpubl.). The data were interpolated over Finland with thin plate spline regression and interpolation functions in R 3.2.5 (functions “Tps” and “interpolate” in R packages fields, Nychka et al. 2016, and raster, Hijmans 2015, respectively; R Core Team 2016) and values at census triangle locations were intersected from the interpolated layer to produce a spruce cone crop estimate for each triangle.

### Weather data

Because weather conditions may affect the behaviour of red squirrels (e.g. Lampio 1967; Pulliainen 1973), weather in the day before the census day was accounted for. The precipitation and mean temperature in the previous day for each triangle were extracted from data provided by the Finnish Meteorological Institute using R packages sp (Pebesma and Bivand 2005) and ncdf4 (Pierce and Pierce 2017). The data sets are available online (at https://avaa.tdata.fi/web/paituli/latauspalvelu) and they display weather parameters for 10 × 10 km grid cells throughout Finland.

### Statistical methods

The effects of immediate and lagged predation pressure, cone abundance, and weather on red squirrel abundance were quantified with a spatiotemporally explicit hierarchical Bayesian model (Cameletti et al. 2013; Jousimo and Ovaskainen 2016) and integrated nested Laplace approximation (INLA) method (Rue et al. 2009). Coordinates were also included as explanatory variables to account for the gradients (e.g. climatic) that run through Finland. All analyses were made using the R environment (R Core Team 2016) and the R-INLA R package (Lindgren and Rue 2015). We assumed the snow-track counts to follow a negative binomial distribution (Jousimo and Ovaskainen 2016), which has parameters for average count rate and overdispersion where we modelled the count rate to be determined by a log-linear combination of the covariates with unknown weights, a spatiotemporal random term, and the triangle lengths as an offset term.

Because track density is defined in continuous space, we modelled the spatial random variation with a Gaussian random field (GRF), whereas the temporal dimension is discrete and is therefore modelled as a Markov process. In specific, spatial dependencies in the GRF are defined by Matérn’s covariance function which includes parameters for spatial range and variance (Lindgren and Rue 2015). Temporal dependencies were modelled by first-order autoregressive process (AR1; Cameletti et al. 2013) and alternatively by first-order random walk (RW1). For computational reasons, the GRF is approximated with a discrete space Gauss–Markov random field (GMRF) by R-INLA. Furthermore, space and time dimensions were assumed independent (separable), although this assumption might not hold. For a more detailed description of the model, see Supporting material.

We ranked different models by the Watanabe–Akaike information criterion (WAIC; Watanabe 2010), which penalizes for model complexity. The best-fit model was selected from the combinations of the covariates: *X*-coordinate, *Y*-coordinate, product of *X*- and *Y*-coordinates, spruce cone abundance, density of pine marten in current and previous year, goshawk kernel height, temperature, precipitation, and triangle type (wildlife or field triangle). The goshawk kernel height in the previous year correlates strongly with that of the next year, and therefore the lagged kernel was omitted. To improve interpretability of the model estimates for covariates in different scales, the numeric covariates were centred to zero and scaled by two standard deviations to be approximately on the same scale with the binary covariate triangle type (Gelman 2008). We compared the models to the following baseline models: an independent negative binomial regression model (non-spatiotemporal model), a spatiotemporal model without covariates (smoothing-only model) and a spatiotemporal model with all covariates. To enable comparison of spatial synchrony between red squirrel populations to that of rodents in other studies, we calculated correlograms using the R package ncf (Bjørnstad 2016). Because many of the site-specific time series contain missing values, we calculated Moran’s I correlograms (Fortin and Dale 2005) for each year separately instead of a multivariate Mantel correlogram.

## Results

### Spatial synchrony

**Fig. 2:**
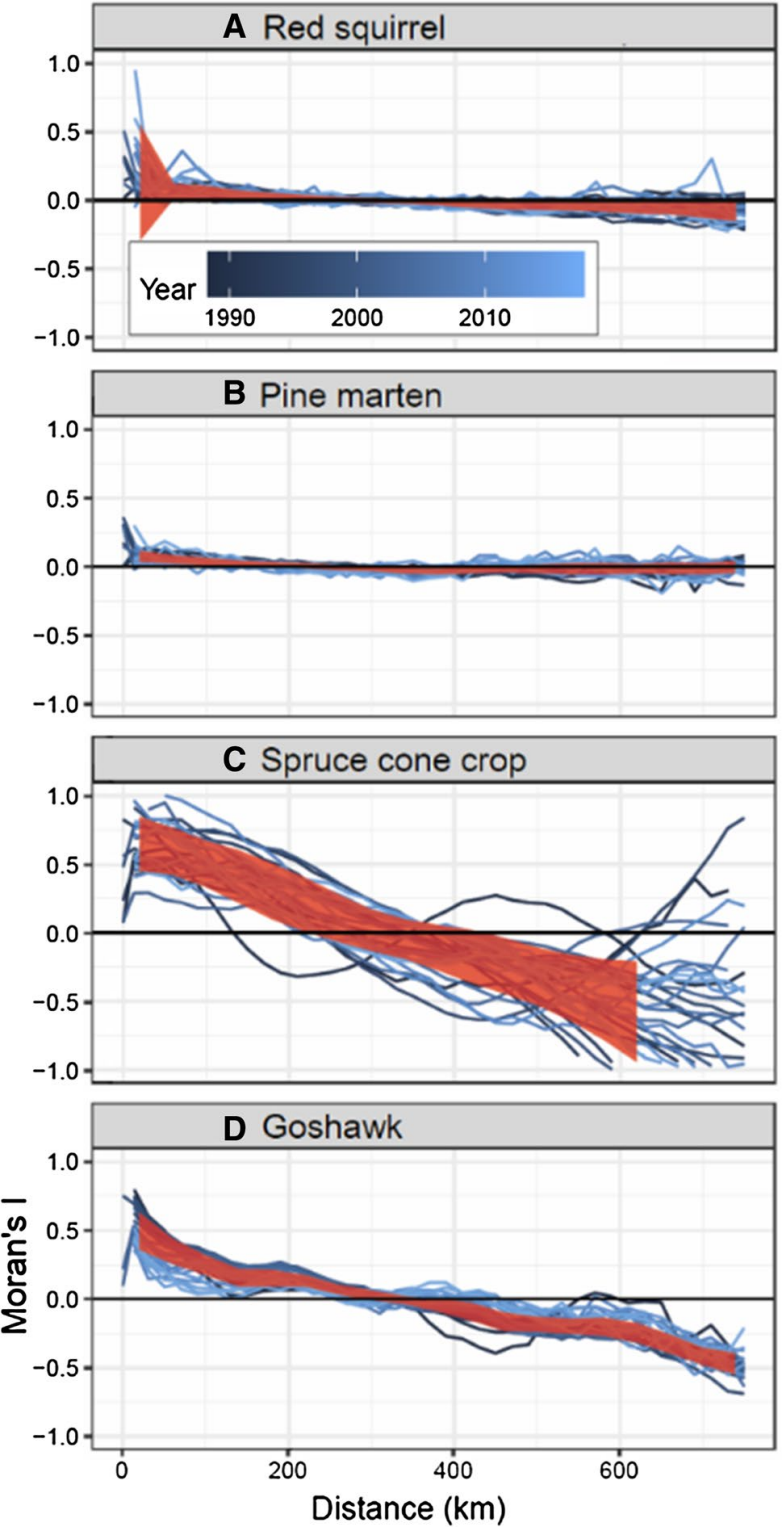
Spatial synchrony of **a** the red squirrel, **b** the pine marten, **c** spruce cone crop and **d** goshawk predation risk index for each distance lag up to 750 km (where the synchronies start to diverge due to low number of samples) and each year as measured by Moran’s I. Each estimate comes from samples in a 20 km bin and the estimates are connected with lines. Ribbon in red shows standard deviation of the correlograms pooled over the years. Black horizontal line at *Y* = 0 indicates no synchrony

The spatial range estimate in the best-fit spatiotemporal model reaches 655 ± 90 km, which is the distance where the spatial autocorrelation becomes negligible between red squirrel populations on average. The long range can be also an indication of spatial nonstationarity and should, therefore, be interpreted with caution. However, also the Moran’s I correlograms show that the synchrony of the red squirrel populations occurs over large spatial scales and abates with distance, except at around 300 km (Fig. 2), where positive autocorrelation turns to negative. The correlograms do not show where the populations become fully disconnected on average as the negative synchrony appears to continue beyond the spatial coverage of the data. So both the spatial range estimate of the best-fit model and the correlograms show that the red squirrel populations are synchronized over very large spatial distances.

The spatial synchrony in the cone crop has more year-to-year variation, and pine marten density shows a mix of weak and no synchrony, while the modelled predation pressure from goshawk shows synchrony similar to that of the red squirrel (Fig. 2). The fluctuation in the number of breeding goshawk pairs between years is highly synchronized over Finland (Ranta et al. 2003), so this result seems reasonable, regardless of the limitations of the data, as discussed in "Goshawk data and kernel construction".

**Fig. 3:**
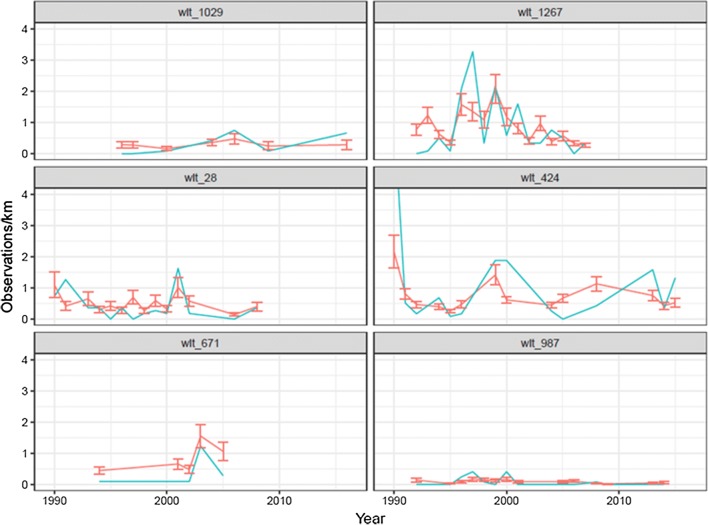
Observed and fitted (± SD) yearly survey counts of red squirrel snow tracks at six randomly selected census sites. Each panel represents a wildlife triangle. Blue line: observed number of red squirrel tracks, red: modelled number of red squirrel tracks

**Fig. 4:**
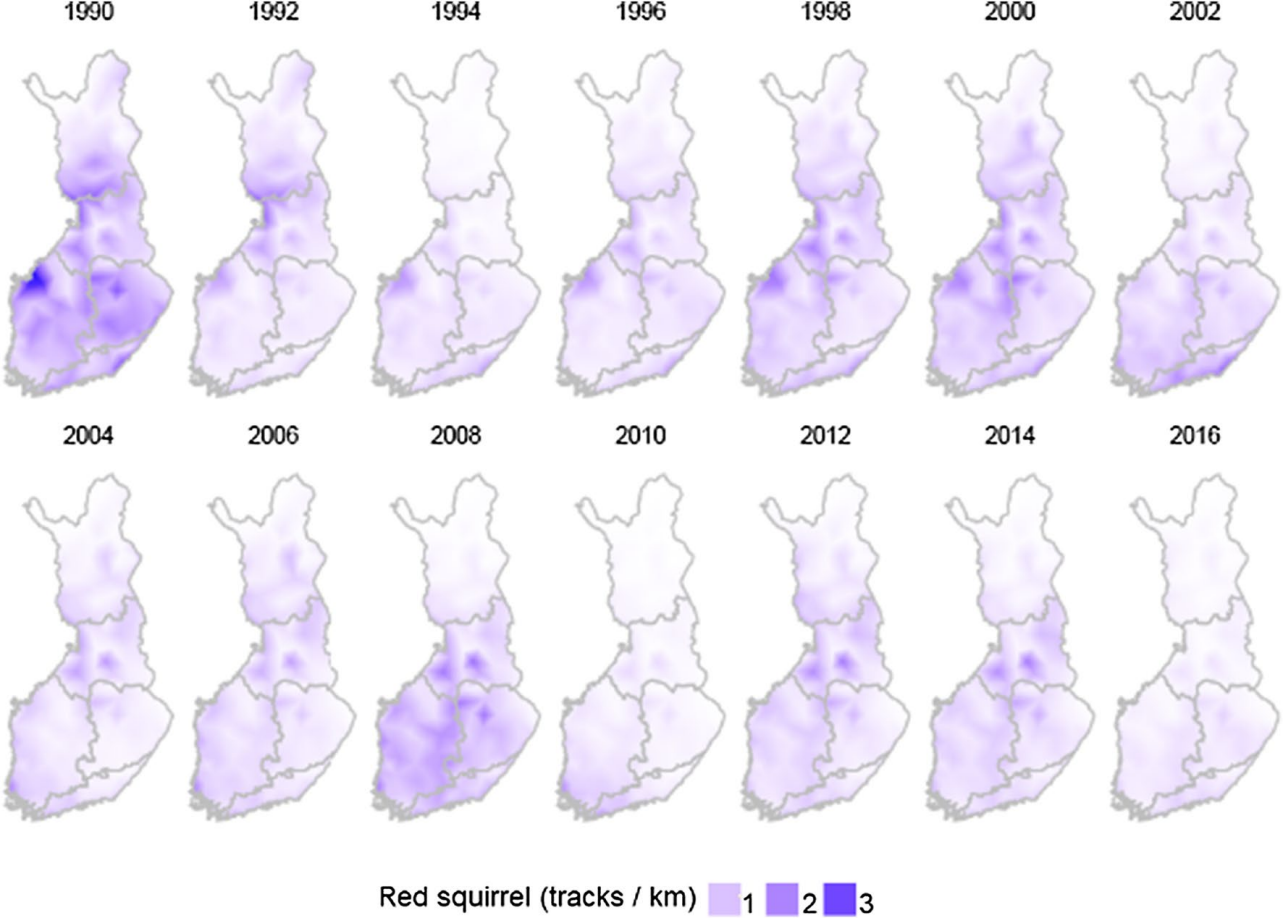
Estimated density (tracks/km of census line) of the red squirrel in Finland 1990–2016 in even years. The triangular artefacts result from the approximation method (see Supplementary material for details)

**Fig. 5:**
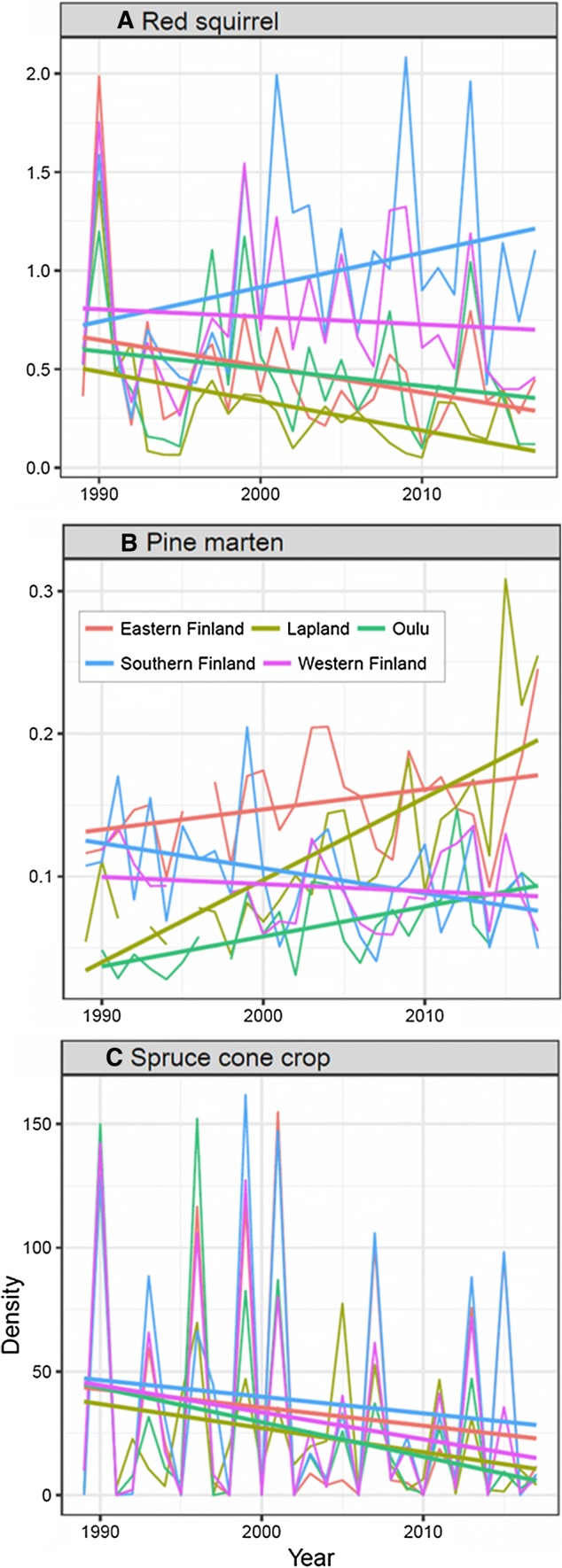
Dynamics of the red squirrel and its food and predators in Finland 1989–2017. Snow-track densities (tracks/1 km of census line) of **a** the red squirrel and **b** the pine marten, and **c** modelled level of previous year’s spruce cone crop (cones per tree) at snow-track census sites in different provinces of Finland. Color legend in (**b**) applies to all panels **a**–**c**

The spatiotemporal model effectively smooths out noise in the observed counts of the snow tracks, which improves accuracy and precision of population density estimates (Jousimo and Ovaskainen 2016), but the spatially aggregated fitted counts still follow closely to the observed (Fig. 3). Spatial residuals generally follow the same pattern each year where south of Finland is overestimated and the north underestimated, which may indicate an insufficient specification of the model to capture the gradient. The resulting spatiotemporal estimates of red squirrel snow-track densities of the best-fit model for every even year are provided in (Fig. 4). The abundance of red squirrels decreases from southwest to northeast (Figs. 4, 5).

### Temporal process

The temporal dependency parameter estimate for the best-fit AR1 model (Model d in Table 1) of 0.99 ± 0.00 means that there is a strong temporal autocorrelation between the observations. The estimate is very close to unity which it cannot reach because the model has been constrained to be stationary. This may indicate that the unknown latent temporal process is a random walk during the study period. However, fit of the RW1 (random walk) model is poor, possibly due to the numerical problems encountered by R-INLA during the estimation of the model (Table 1).

**Table 1:** The best-fit spatiotemporal (ST) models for autoregressive (AR) and random walk (RW) models and baseline models, of which model d is the best one

Model		WAIC
a	Independent, pine marten + goshawk + cones + triangle type + temperature + rain + *XY*	59,171
b	ST, AR1, no covariates	56,767
c	ST, AR1, pine marten + goshawk + cones + triangle type + temperature + rain	56,450
d	ST, AR1, pine marten + goshawk + cones + triangle type + temperature + rain + *XY*	**56,449**
e	ST, RW1, pine marten + goshawk + cones + triangle type + temperature + rain + *XY*	60,525

Ranking is by the Watanabe–Akaike information criterion (WAIC) where lower score indicates better fit model d is the best one (in bold)

Red squirrel track density has increased in Southern Finland but decreased in other parts of the country (Fig. 5). The spruce cone crop shows high year-to-year fluctuations (Fig. 5). The pine marten has an increasing linear trend over the study period, but mixed trends on the provincial level (Fig. 5). There appear to be similar mixed provincial trends for the modelled predation risk from goshawk, but the search effort has not been constant over time and space, and therefore trends in the goshawk data are not reliable.

### Drivers of red squirrel abundance

**Fig. 6:**
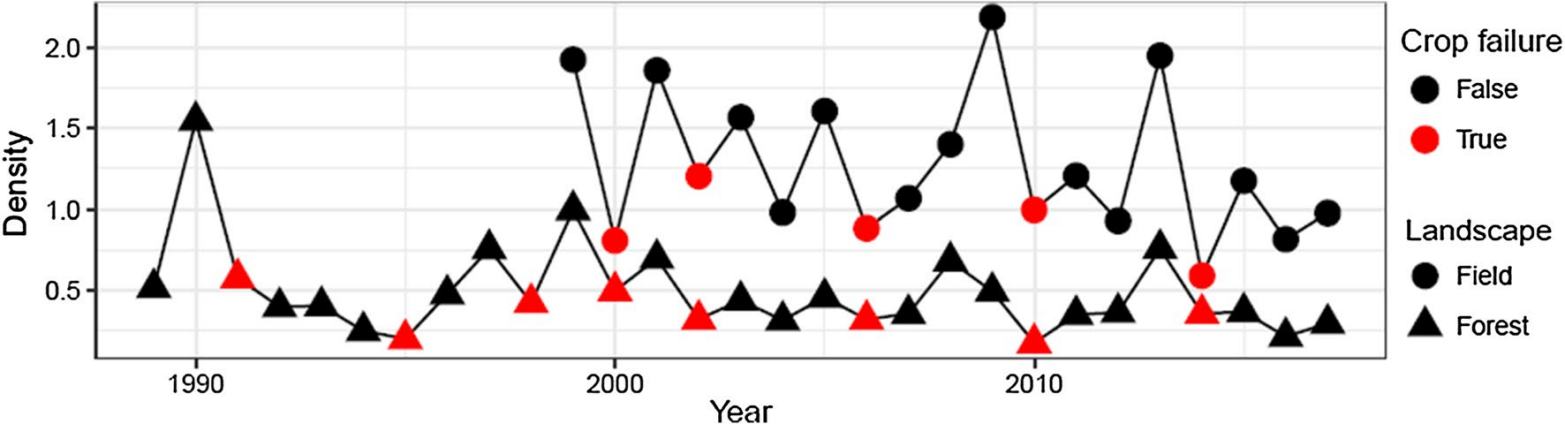
Red squirrel densities (tracks/km) in forest and field landscapes (i.e. in wildlife triangles and field triangles). Red symbols indicate spruce cone crop failures in the preceding year

The best model fit was achieved with the combination of covariates pine marten density, goshawk kernel height, spruce cone index, census landscape (triangle) type, precipitation, temperature, and the product of *X* and *Y*-coordinates (Model d in Table 1). However, variance estimate of the *X*-coordinate is suspiciously high and we therefore hereafter consider the model without coordinates which had the second highest WAIC (Model c in Table 1). The pine marten, the goshawk, and the cone index were positively associated with the red squirrel snow-track density (Tables 2). In contrast, landscape type had a significant negative effect, i.e. snow-track density was greater in field than in forest triangles (Fig. 6; Table 2).

**Table 2:** Estimates from hierarchical Bayesian model for factors driving red squirrel population density: mean, standard deviation, 2.5% quantile, median, 97.5% quantile and mode of weights of the effects on red squirrel census density

	Mean	SD	2.5% Q	Median	97.5% Q	Mode
Intercept	− 1.73	1.82	− 5.40	− 1.71	1.87	− 1.69
Pine marten	0.22	0.02	0.17	0.22	0.27	0.22
Goshawk	0.11	0.04	0.03	0.11	0.18	0.11
Spruce cone crop	0.37	0.07	0.23	0.37	0.51	0.37
Triangle type (wildlife/field)	− 0.57	0.06	− 0.69	− 0.57	– 0.46	− 0.57
Temperature	0.30	0.03	0.25	0.30	0.36	0.30
Precipitation	− 0.24	0.03	− 0.30	− 0.24	-0.19	− 0.24

The data were scaled relative to each other so that estimates can be directly compared

There were more snow tracks when temperature was higher and precipitation lower. The estimated weights of the covariates are approximately relative to each other in strength (Table 2). Landscape type has the largest absolute effect and cone index has the strongest positive effect of all covariates on red squirrel density. Spruce cone crop failures seem to be mostly, but not solely, responsible for the drops in red squirrel track counts (Fig. 6).

## Discussion

The descriptive analysis and the spatiotemporal model results show that the abundance of the red squirrel is autocorrelated both spatially and temporally. Comparison of the best-fit model to the baseline models demonstrates that the spatiotemporal model explains variation in the red squirrel snow-track abundance better than the non-spatiotemporal one, and that the selected covariates in the best-fit model improve model performance compared to the full model or the smoothing model without covariates.

### Spatial synchrony

The estimated spatial range of the synchrony in red squirrel populations of ca. 655 km is remarkably large for a small mammal. Methodological differences can hinder comparison of synchrony estimates between studies, which is why we also calculated Moran’s I, a commonly used metric of spatial autocorrelation or synchrony, in addition to the spatial range estimate from the spatiotemporal model. Most previous studies where some sort of correlograms have been calculated from data on small rodents have yielded estimates that are an order of magnitude smaller, being ca. 30–50 km (e.g. Steen et al. 1996; Bjørnstad et al. 1999). However, red squirrels are arguably more mobile and disperse much further than, for example, voles (Lambin 1994; Lurz et al. 1997; Fey et al. 2016), which accounts for some of the range of the synchrony. Still, such a large range hints on an underlying mechanism that also operates on a large scale. Indeed, climate-driven synchrony can occur on a scale of hundreds of kilometres, or well up to 1000 km (e.g. Post and Forchhammer 2002). Abiotic factors, mainly temperature, which affect the cone crop, seem like a plausible explanation for the synchrony in red squirrel population fluctuations occurring on a scale of hundreds of kilometres. The synchrony in the cone crop occurs over long distances as well, as shown by the correlograms, and in the case of spruce cone crop is mostly driven by temperature (Ranta et al. 2010; Pukkala et al. 2010, for other factors than weather behind a tree mast see e.g. Bogdziewicz et al. 2019). The fairly uniform species composition in Finnish boreal forests is also a possible explanation for the large spatial range of the synchrony observed in this study. In forest systems with high tree species diversity and several potential food resources for herbivores and granivorous, the synchronization effect is likely to be less pronounced.

Of the possible mechanisms causing synchrony, i.e. dispersal (Ranta et al. 1995), nomadic predators (Norrdahl and Korpimäki 1996; Korpimäki et al. 2005), trophic interactions with another species that also shows synchrony (Byholm et al. 2002; Satake et al. 2004; Cattadori et al. 2005), and Moran effect (Moran 1953; Royama 1992), the two latest seem to be acting here. In other words, red squirrel populations are synchronized because weather, a type of Moran effect, causes their food resources to be synchronized over a large scale. Also Ranta et al. (1997) demonstrated how the Moran effect could lead to synchrony between red squirrel populations. Similarly, Kemp and Keith (1970) showed, based on fur records, how the population fluctuations of the North American red squirrel (*Tamiasciurus hudsonicus*) are synchronized over vast areas following food availability and weather.

### Temporal processes

The strong temporal autocorrelation dependency suggests that the fluctuations in the red squirrel populations are nonstationary, although previous studies have concluded that red squirrel numbers generally fluctuate around an equilibrium density (Wauters et al. 2004, 2008). The data suggest a slightly decreasing trend for red squirrels throughout the study area, except for Southern Finland. A recent large-scale study from partially the same snow-track data (Turkia et al. 2018b) as well as a diet analysis of red squirrel’s predators (Selonen et al. 2010) also suggested that the red squirrel has been declining. Turkia et al. (2018b) showed that warming of winters is one reason behind the decline, but also that there are other, unknown reasons that were not found. In Finland, one possible reason that can have partially decreased the density of the red squirrel population in addition to the climate change is too heavy forest management, which has decreased the mean age of forests. Given that mature and old trees produce more cones than young trees, it could be that Finnish forests support fewer seed predators than before. The global trend is that even least concern species are declining worldwide (Ceballos et al. 2017) and also in Finland, where most of the threatened species live in forests (Hyvärinen et al. 2019).

### Bottom-up and top-down effects

The simultaneous analysis of the effects of predators and food show a strong bottom-up effect on red squirrel track density with no indication of top-down negative effects on population level. The clear positive effect of cone crop abundance on red squirrels was expected, because it is well established in the literature that red squirrel numbers follow fluctuations in cone crop (e.g. Andrén and Lemnell 1992; Wauters et al. 2008; Selonen et al. 2015). The density of red squirrel snow tracks also positively correlated with that of its predators, the pine marten and the goshawk. This association is probably caused by shared habitat preferences rather than any biotic interaction between the species, which should result in a negative relationship. Both the red squirrel and the predators prefer mature coniferous forest (Andrén and Delin 1994 on red squirrels; Storch et al. 1990 on pine martens; Tornberg et al. 2006 on goshawk), so considering the fragmented nature and high level of intensive management of the Finnish forests, it is not surprising that they are found in abundance in the same census sites.

Alternatively, it could be argued that predators seem to have won the predator–prey space race, where predators try to maximize spatial overlap with their prey (Sih 2005). Red squirrels need to settle in forests with enough mature coniferous trees that provide them with food, whereas predators can settle where their prey are. Suitable habitat in terms of food availability could thus be viewed as a spatial anchor (Sih 2005) which forces red squirrels to settle where their resources are, regardless of possible predation risk. Interestingly, also Sheehy and Lawton (2014) found red squirrel and pine marten occurrences to be positively correlated, but this was in Ireland, where the invasive grey squirrel (*Sciurus carolinensis*) has replaced the red squirrel in many places, and recovery of red squirrels appears to be facilitated by pine martens preying upon grey squirrels more than red squirrels. The positive spatial association of the red squirrel with its predators supports earlier results that even though red squirrels sometimes form a substantial proportion of goshawks’ and pine martens’ diets, depending on availability of other prey types (Widén 1987; de Marinis and Masseti 1995; Penteriani 1997; Pulliainen and Ollinmäki 1996; Tornberg et al. 2006; Selonen et al. 2010), red squirrel populations are not suppressed by predation (Gurnell 1983 cited in Petty et al. 2003; Sheehy and Lawton 2014; but see Halliwell 1997; Selonen et al. 2016a). The absence of lagged effects, i.e. negative effects between the previous years’ pine marten abundance and the red squirrel abundance in later years, further supports the view that predation does not shape the large-scale population dynamics of the red squirrel (see also e.g. White 2013).

It has to be noted, however, that the red squirrel has increased in the only part of Finland where both the pine marten and the modelled goshawk predation risk have decreased based on the current study, that is, in Southern Finland. This may still indicate a regulatory role of the predator guild as a whole on red squirrel populations, while there are also a number of other possible reasons behind these trends. For example, urbanization and a mosaic-like habitat structure may play a role, as Southern Finland is the most densely populated part of Finland. Red squirrels do well in human-dominated areas (e.g. Fey et al. 2016, Jokimäki et al. 2017, Turkia et al. 2018a), where they find alternative food resources provided by humans. Pine martens have not yet settled to urban areas in Finland, and goshawks mainly visit urban areas in the winter (Vuorisalo et al. 2003), although goshawks have recently also started to thrive near to and within cities (Solonen 2008). However, the goshawk has declined in Finland and was listed as near-threatened in the latest Red Lists of Finnish birds (Tiainen et al. 2016; Lehikoinen et al. 2019) due to habitat loss, i.e. loss of old-growth forests.

Previous studies suggest that the effect of goshawks on red squirrels is not straightforward, but may vary depending on other factors than mere abundances of predator and prey. For example, Turkia et al. (2018a) found support for habitat-specific predation by goshawks, whereby goshawks affected red squirrel occurrence most negatively in a mutually non-preferred habitat. Locally, predator avoidance can affect space use of red squirrels, but on a larger scale, common habitat preferences arise as the dominant phenomena causing positive correlation between their abundances.

### Landscape type

There were more red squirrel tracks in the field triangles than in the wildlife triangles. Despite their name, the field triangles are not completely on fields, but by definition, about half of the census line should be on fields (from 25 to 67%), and the rest runs through forests and built-up areas, such as townships, villages and farmsteads (Luke 2018), reflecting the mosaic-like landscape typical of southern and western Finland. Most of the red squirrel snow tracks are in the forested parts and close to human settlements and not in the open field parts of field triangles (J. Tiainen unpubl.). As for example Jokimäki et al. (2017), Dylewski et al. (2016) and Turkia et al. (2018a) showed, the red squirrel thrives close to forest edges and human settlements, which is a likely reason for the high density of tracks in the field triangles. Alternative food resources provided by humans are important especially when the spruce cone crop is low. Furthermore, the pine marten does not usually come so close to human settlements as the red squirrel does. Indeed, the density of pine marten tracks was significantly lower in the field than in the forest triangles in the same area. It may be that the increasing trend in Southern Finland, as opposed to the declining trend in the other parts of the country, is also linked to the location of the field triangles in this part of Finland. The red squirrel seems to be declining in forests (Turkia et al. 2018b), but the dynamics in more mosaic-like areas may be different. The movement between close forest patches and from forests to yards in a mixed landscape is also consistent with the view that red squirrels can tolerate moderate habitat fragmentation (reviewed in Selonen and Hanski 2015).

## Conclusions

Our results show that red squirrel populations are highly spatially synchronized over an exceptionally large distance. The synchrony seems to be driven by food abundance and ultimately weather, while the red squirrel’s main predators, the pine marten and the goshawk, do not seem to have a negative effect. This study thus suggests that the synchrony is driven by fluctuating resources, and demonstrates how an explicit spatiotemporal approach can improve model performance for fluctuating populations. We also found that the red squirrel has declined in most parts of Finland for 29 years (see also Turkia et al. 2018b). This is an important conservation message that highlights the urgent need for understanding the underlying causes behind population dynamics of the species.

## Electronic supplementary material

Below is the link to the electronic supplementary material.

Supplementary file1 (PDF 182 kb)
