# Supplementary material for: Large-scale spatial synchrony in red squirrel populations driven by a bottom-up effect
Source: Oecologia. 2020 Jan 11;192(2):425–37. doi: 10.1007/s00442-019-04589-5 (PMC7002333; doi:10.1007/s00442-019-04589-5)
Supplement: Supplementary file 1 — Supplementary file1 (PDF 182 kb) [file 442_2019_4589_MOESM1_ESM.pdf]

Large-scale spatial synchrony in red squirrel populations driven by bottom-up effects

Jousimo J[a*], Turkia T[b*+], Rintala J[c], Valkama J[d], Hokkanen T[e], Selonen V[b]

[a] Metapopulation Research Centre, University of Helsinki, Finland

[b] Department of Biology, University of Turku, Finland

[c] Natural Research Institute Finland, Finland

[d] Finnish Museum of Natural History, Finland

[*] These authors contributed equally to this work.

[+] Corresponding author: tysisit@utu.fi, orcid.org/0000-0002-2098-8402

Supplementary material

Spatio-temporal model specification

Let y_{st} be the snow track counts of red squirrels observed at census site $s \in S$ in year $t \in T$ and L_s the length of the census transect. Furthermore, let $x_{1st}, \ldots, x_{pst}$ be the p explanatory variables such as spruce cone crop yield, pine marten density, etc. Expected count of the red squirrels is given by $\mu_{st} = \mathrm{E}(y_{st} \mid x_{1st}, \ldots, x_{pst})$, and since μ_{st}/L_s is the expected census density (counts/km), we define

$$\log(\mu_{st}) = \log(L_s) + \beta_0 + \beta_1 x_{1st} + \beta_2 x_{2st} + \ldots + \beta_p x_{pst} + z_{st},$$

where $\log(L_s)$ is an offset term and z_{st} is a spatio-temporal residual. It is assumed that the counts y_{st} come from a negative binomial distribution with parameters μ_{st} and dispersion α, which allows more relaxed assumption of the mean–variance relationship than the Poisson distribution (White and Bennet 1996).

Assuming that the residuals are jointly Gaussian, estimation of the residuals and model parameters with an iterative method requires inverting the variance–covariance matrix with $|S|^2|T|^2$ elements at each step, which quickly becomes computationally very expensive. Therefore the residuals are approximated with a linear transformation $z_{st} = \sum_{v \in V} \boldsymbol{A}_{sv} \boldsymbol{\xi}_t$ which maps the latent Gaussian field $\boldsymbol{\xi}_t = [\xi_{1t}, \ldots, \xi_{vt}]^\top$ from a lower rank space of fixed locations $v \in V$ to the space of the observation locations. In specific, the sparse matrix $\boldsymbol{A}_{sv} = 1$ if latent location v is mapped to s, otherwise $\boldsymbol{A}_{sv} = 0$ (Cameletti et al. 2013). Granularity of the approximation is controlled by the number of the latent locations $|V| < |I|$ and chosen by the available computational resources. We found the latent locations V from a triangulation over the study area which was extended beyond to avoid edge effects (Lindgren, Rue, and Lindström 2011) (Fig. 1). The resulted mesh contains $|V| = 522$ latent locations compared to the number of unique sites $|S| = 1460$ in the census data.

The temporal dependencies in the latent residuals are assumed to follow a stationary autoregressive process with a single lag, $\xi_{st} = \phi \xi_{s,t-1} + \epsilon_{st}$, where $|\phi| < 1$ is a parameter controlling the degree of temporal dependency. Temporal residual vector $\boldsymbol{\xi}_t$ has a zero-centered Gaussian structure with the variance–covariance matrix $\boldsymbol{\Sigma}_T$, where $\mathrm{Cov}(\epsilon_{st}, \epsilon_{st'}) = \phi^{|t-t'|}\sigma^2/(1-\phi^2)$ for $1 \leq t, t' \leq T$. Moreover, spatial latent residuals ϵ_{st} are Gaussian as well with covariates $\mathrm{Cov}(\epsilon_{st}, \epsilon_{s't}) = \sigma^2 C(d_{ss'}, \kappa, \nu)$ for $s \neq s'$ and σ^2 on the diagonal of the variance–covariance matrix $\boldsymbol{\Sigma}_S$. Function C is of the Matérn class modelling the autocovariance as a function of distance $d_{ss'}$ between two sites with parameters $\kappa > 0$ for the range of spatial synchrony and ν for smoothness of the autocovariance fixed to $\nu = 2$ (Lindgren, Rue, and Lindström 2011). Spatial and temporal dimensions are assumed to be independent, hence $\mathrm{Cov}(\xi_{st}, \xi_{s't'}) = 0$ for all $s \neq s'$, $t \neq t'$.

Since $\boldsymbol{\Sigma}_S$ is a dense matrix, it is approximated with a sparse precision matrix $\boldsymbol{Q}_S$ using the stochastic partial differential equation approach described in Lindgren, Rue, and Lindström (2011). Essentially, the

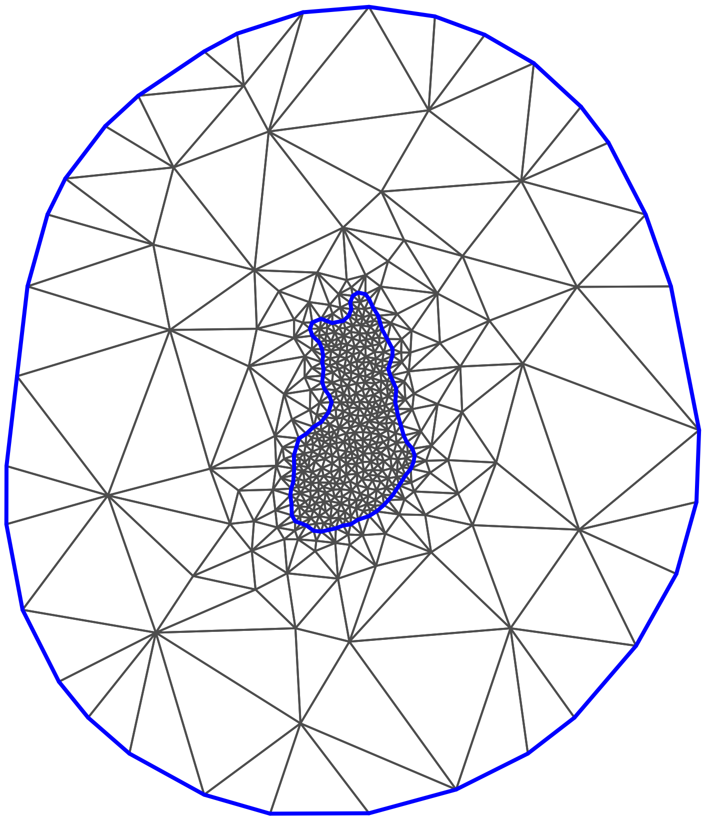

Figure 1: A mesh of latent locations (at the mesh nodes) to approximate the red squirrel density at the observation locations. Inner blue line indicates the study area and the outer the extended area.

continuous spatial surface is approximated with a discrete representation which is an irregular lattice and has the properties of Gaussian Markov random field (GMRF) (Rue and Held 2005). Inverse of $\boldsymbol{\Sigma}_T$ can be found analytically and is sparse (Cameletti et al. 2013). Since the assumption of no space-time interactions, the precision matrices can be combined to a spatio-temporal precision matrix with Kronecker product $\boldsymbol{Q} = \boldsymbol{Q}_T \otimes \boldsymbol{Q}_S$. Discrete representation of the spatio-temporal dependencies between the latent residuals embedded in the sparse precision matrix $\boldsymbol{Q}$ enables the use of fast linear algebra algorithms to operate on it (Rue and Held 2005).

The unknowns are inferred from a Bayesian hierarchical model with the joint posterior distribution

$$p(\boldsymbol{\theta}, \boldsymbol{\psi} \mid \boldsymbol{y}) \propto p(\boldsymbol{\psi}) p(\boldsymbol{\theta} \mid \boldsymbol{\psi}) \prod_{t \in T} p(\boldsymbol{y}_t \mid \boldsymbol{\theta}, \boldsymbol{\psi})$$

including the parameters $\boldsymbol{\theta} = [\boldsymbol{\beta}, \boldsymbol{\xi}]$ and hyperparameters $\boldsymbol{\psi} = [\alpha, \phi, \sigma^2, \kappa, \nu]$ (Rue, Martino, and Chopin 2009). The observations are conditionally independent following the negative binomial distribution $\boldsymbol{y}_t \mid \boldsymbol{\theta}, \boldsymbol{\psi} \sim NB(\boldsymbol{\beta}^\top \boldsymbol{x}_t + \boldsymbol{A}\boldsymbol{\xi}_t, \alpha)$ and the latent residuals are assigned with the GMRF prior $\boldsymbol{\xi} \mid \boldsymbol{\psi} \sim N(\boldsymbol{0}, \boldsymbol{Q}^{-1})$. Posterior marginal estimates are found for the parameters from

$$p(\boldsymbol{\theta}_i \mid \boldsymbol{y}) = \int p(\boldsymbol{\psi} \mid \boldsymbol{y}) \int p(\boldsymbol{\theta} \mid \boldsymbol{y}, \boldsymbol{\psi}) \mathrm{d}\boldsymbol{\theta}_{-i} \mathrm{d}\boldsymbol{\psi}$$

and for the hyperparameters from

$$p(\boldsymbol{\psi}_j \mid \boldsymbol{y}) = \int p(\boldsymbol{\psi} \mid \boldsymbol{y}) \mathrm{d}\boldsymbol{\psi}_{-j}.$$

Since computing these integrals is analytically intractable in general, we estimated them with the integrated nested Laplace approximation method (Rue, Martino, and Chopin 2009) implemented in the R-INLA R-package (Lindgren and Rue 2015). The regression coefficients $\boldsymbol{\beta}$ and hyperparameters $\boldsymbol{\psi}$ were all assumed independent of each other and assigned with the default priors provided by the R-INLA software.

References

Cameletti, Michela, Finn Lindgren, Daniel Simpson, and Håvard Rue. 2013. "Spatio-Temporal Modeling of Particulate Matter Concentration Through the Spde Approach." *AStA Advances in Statistical Analysis* 97 (2): 109–31. doi:10.1007/s10182-012-0196-3.

Lindgren, Finn, and Håvard Rue. 2015. "Bayesian Spatial Modelling with R-Inla." *Journal of Statistical Software* 63 (19). doi:10.18637/jss.v063.i19.

Lindgren, Finn, Håvard Rue, and Johan Lindström. 2011. "An Explicit Link Between Gaussian Fields and Gaussian Markov Random Fields: The Stochastic Partial Differential Equation Approach." *Journal of the Royal Statistical Society: Series B (Statistical Methodology)* 73 (4). Blackwell Publishing Ltd: 423–98. doi:10.1111/j.1467-9868.2011.00777.x.

Rue, Håvard, and Leonhard Held. 2005. *Gaussian Markov Random Fields: Theory and Applications*. Chapman & Hall/CRC.

Rue, Håvard, Sara Martino, and Nicolas Chopin. 2009. "Approximate Bayesian Inference for Latent Gaussian Models by Using Integrated Nested Laplace Approximations." *Journal of the Royal Statistical Society: Series B (Statistical Methodology)* 71 (2): 319–92.

White, Gary C., and Robert E. Bennet. 1996. "Analysis of Frequency Count Data Using the Negative Binomial Distribution." *Ecology* 77 (8): 2549–57.
